# Still waters run deep: progression of left ventricular mass and stagnant atherosclerosis of carotid artery in patients with type 2 diabetes mellitus and coronary heart disease - insights from a long-term CMR study

**DOI:** 10.1186/1532-429X-16-S1-P85

**Published:** 2014-01-16

**Authors:** Tingting Xiong, David Winkel, Eike Nagel, Nikolaus Tilling, Jan-Hendrik Hassel, Ashraf Hamdan, Rolf Gebker, Eckart Fleck, Ursula Plöckinger, Sebastian Kelle

**Affiliations:** 1 Cardiology, German Heart Institute Berlin, Berlin, Germany; 2 Division of Imaging Sciences and Medical Engineering, Kings College London, London, UK; 3 Interdisziplinäres Stoffwechsel-Centrum, Campus Virchow-Klinikum, Charité-Universitätsmedizin Berlin, Berlin, Germany; 4 Cardiology, Chaim Sheba Medical Center, Tel Hashomer, Sackler Faculty of Medicine, Tel-Aviv University, Tel Aviv, Israel

## Background

Patients with type 2 diabetes mellitus (T2DM) and coronary artery disease (CAD) are at high cardiovascular risk. Both carotid intima-media thickness (CIMT) and left ventricular (LV) mass are predictors for cardiovascular events and are commonly increased in these patients. However, the mechanism of association is poorly understood. This study sought to determine that relationship and to evaluate the role of CIMT in risk prediction in T2DM patients with and without CAD.

## Methods

Cardiovascular magnetic resonance (CMR) imaging was performed at baseline and at 2-year follow-up in 88 patients with type 2 diabetes (52 men, mean age 61 ± 8 years) including 31 patients with CAD. LV mass index (LVMI) was determined by indexing LV mass to body surface area. For carotid arteries lumen area and total vessel area were assessed and vessel wall area and vessel wall ratio (VWR = vessel wall area/body surface area) were calculated. CIMT was measured by ultrasound.

## Results

**Figure 1:**
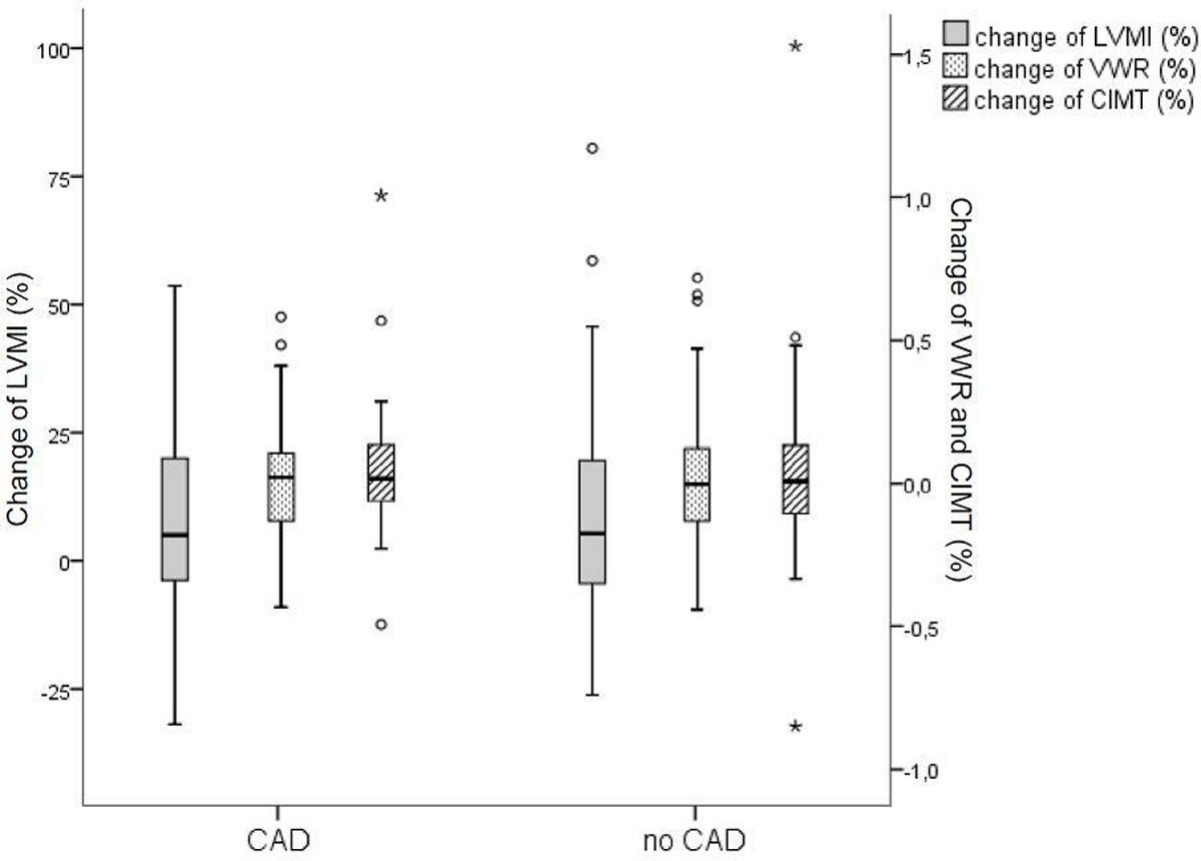
**Boxplot showing the relative change of left ventricular mass index (LVMI), Vessel Wall Ratio (VWR) and carotid intima-media thickness (CIMT) for patients with and without coronary heart disease (CAD)**.

All patients were under optimal medical treatment. Patients with and without CAD had similar age (62 ± 8 vs. 60 ± 9 years, p = 0.217) and duration of T2DM (12.6 ± 7.2 vs. 10 ± 8 years, p = 0.133). Patients with CAD showed a higher LVMI at both baseline (67.42 ± 13.21 vs. 57.75 ± 12.87, p = 0.001) and follow-up (72.43 ± 17.27 vs. 62.47 ± 15.14 g/m2, p = 0.006) compared to patients without CAD. After 2 years, both groups had a significant higher LVMI (CAD: p = 0.031; no CAD: p = 0.002), but showed no difference in the relative increment (p = NS) (see Figure 1). At carotid level, VWR at baseline was higher in patients with CAD (22.1 ± 7.4 vs. 19.2 ± 5.6, p = 0.04), but showed no change in both groups. CIMT also remained unchanged and showed no difference between patients with and without CAD at both examinations. The two groups did not differ in relative change of VWR and CIMT (p = NS) (see Figure 1).

## Conclusions

With higher values in CAD patients at baseline, LVMI showed a significant increment in T2DM patients with and without CAD. Interestingly, both vessel wall ratio and CIMT of carotid artery remained unchanged and showed no difference between the subgroups. These findings suggest that traditional CIMT assessment for risk stratification may underestimate risk in T2DM patients especially with co-existing CAD. A combined cardiac and vascular imaging approach may improve risk prediction in this patient group.

## Funding

None.

